# Supplementary material for: Biodegradable Mulch Films and Bioformulations Based on Trichoderma sp. and Seaweed Extract Differentially Affect the Metabolome of Industrial Tomato Plants
Source: J Fungi (Basel). 2024 Jan 25;10(2):97. doi: 10.3390/jof10020097 (PMC10890107; doi:10.3390/jof10020097)
Supplement: Supplementary file 1 [file jof-10-00097-s001.zip › jof-2809596-supplementary.pdf]

Supplementary material

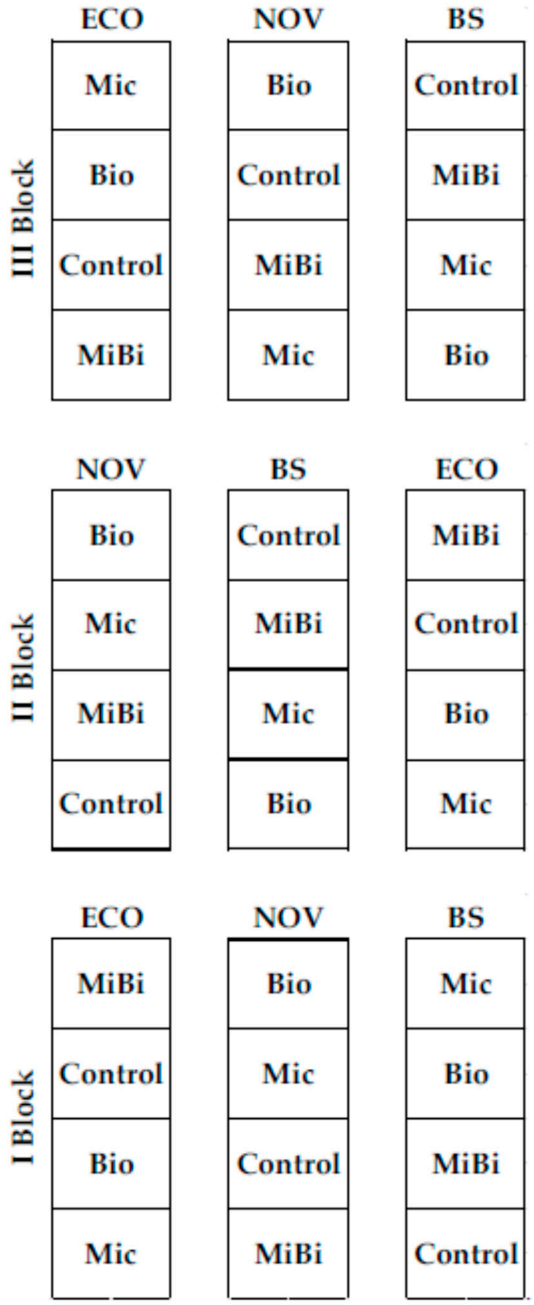

Figure S1. Layout of the experimental design (Di Mola et al., 2023).

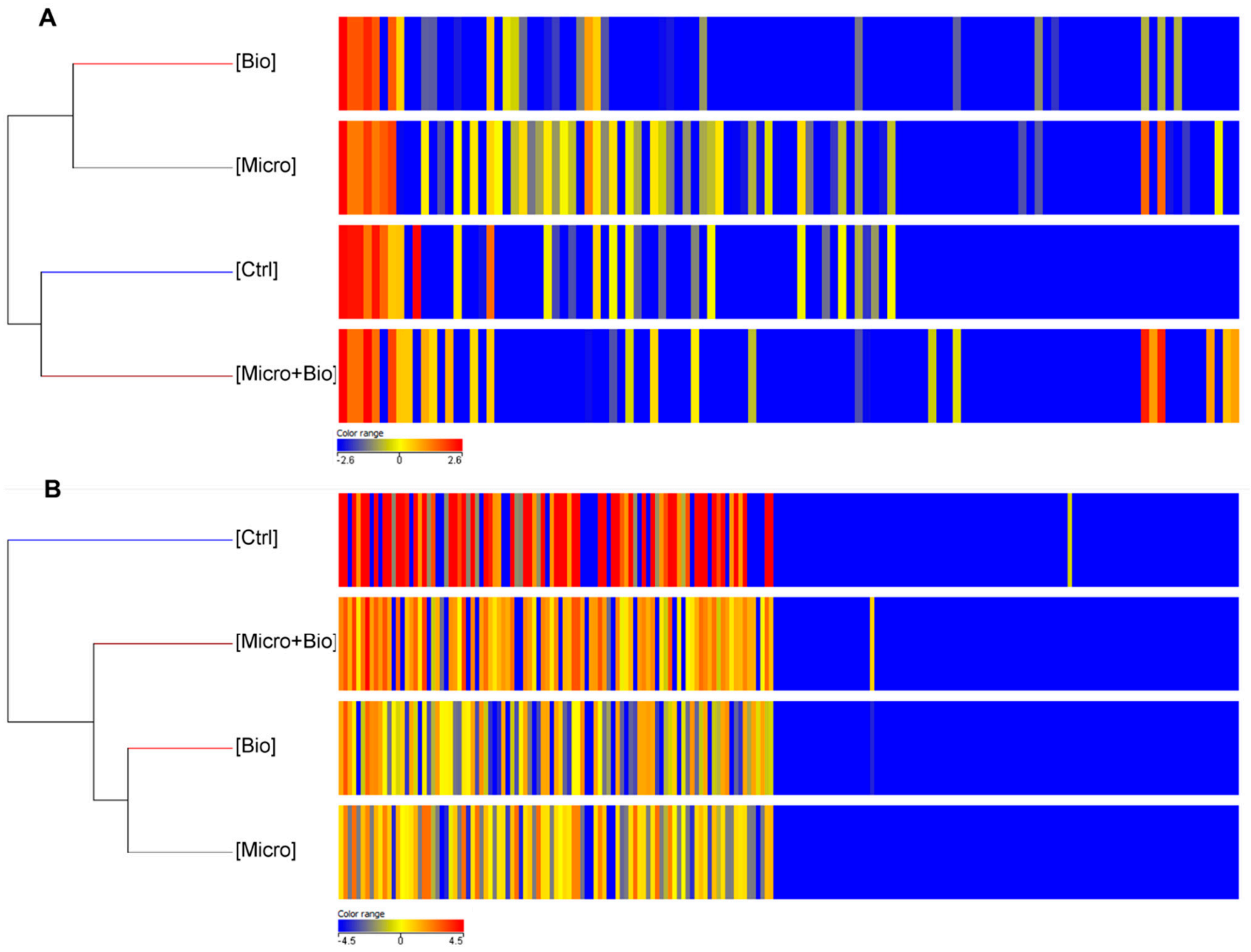

Figure S2. Heat map and dendrograms obtained by comparing the differential metabolomic profiles of tomato berries upon treatments with biostimulants, compared to plants treated with water only (Ctrl). The range of colors from blue to red shows how the intensities of the different compounds vary from the least abundant to the most abundant respectively. **A:** Differential metabolomic profiles obtained in positive ionization mode (ESI+). **B**: Differential metabolomic profiles obtained in negative ionization mode (ESI-). Each column represents a metabolite, while data for each treatment is presented in rows. The heat map was developed using MassProfiler Professional bioinformatics software (Agilent Technologies) and statistical differences were determined by one-way ANOVA test ($p < 0.05$).

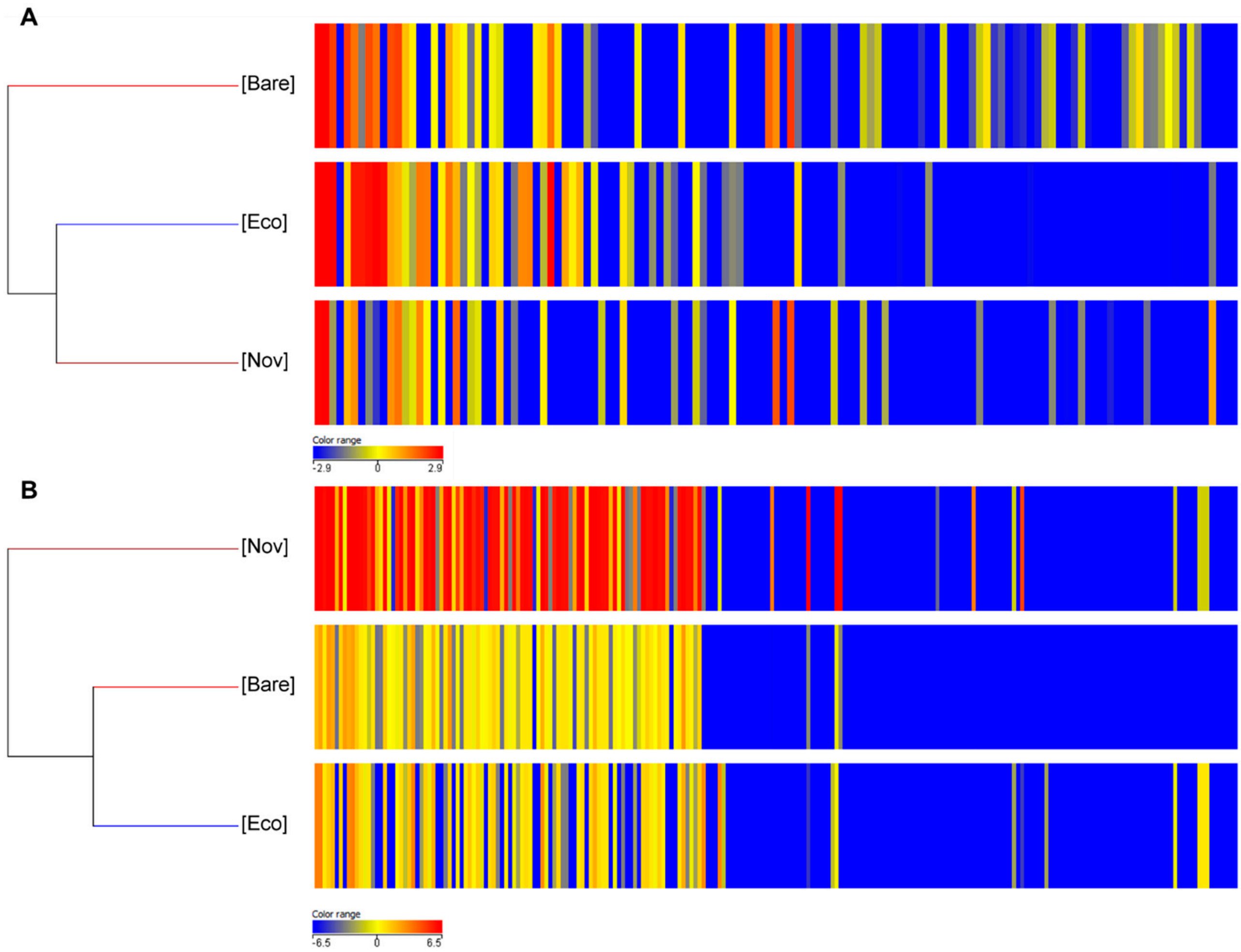

Figure S3. Heat map and dendrograms obtained by comparing the differential metabolomic profiles of tomato berries cultivated on mulch biofilms and upon treatments with T22, compared to plants cultivated on bare soil and treated with T22. The range of colors from blue to red shows how the intensities of the different compounds vary from the least abundant to the most abundant respectively. **A**: Differential metabolomic profiles obtained in positive ionization mode (ESI+). **B:** Differential metabolomic profiles obtained in negative ionization mode (ESI-). Each column represents a metabolite, while data for each treatment is presented in rows. The heat map was developed using MassProfiler Professional bioinformatics software (Agilent Technologies) and statistical differences were determined by one-way ANOVA test ($p < 0.05$).

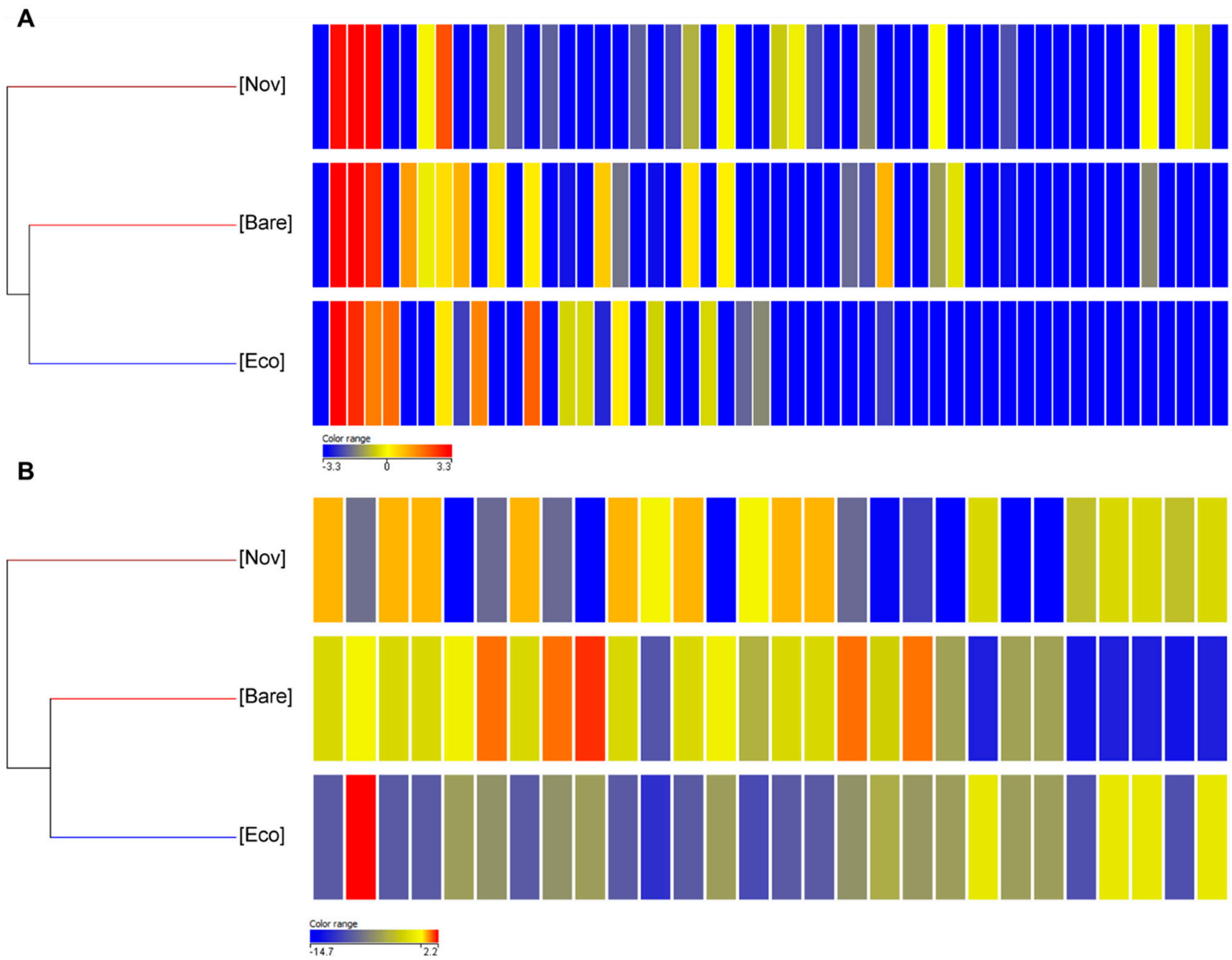

Figure S4. Heat map and dendrograms obtained by comparing the differential metabolomic profiles of tomato berries cultivated on mulch biofilms and upon treatments with the combination of both biostimulants, compared to plants cultivated on bare soil and treated with the combination of both biostimulants. The range of colors from blue to red shows how the intensities of the different compounds vary from the least abundant to the most abundant respectively. **A**: Differential metabolomic profiles obtained in positive ionization mode (ESI+). **B**: Differential metabolomic profiles obtained in negative ionization mode (ESI-). Each column represents a metabolite, while data for each treatment is presented in rows. The heat map was developed using MassProfiler Professional bioinformatics software (Agilent Technologies) and statistical differences were determined by one-way ANOVA test ($p < 0.05$).

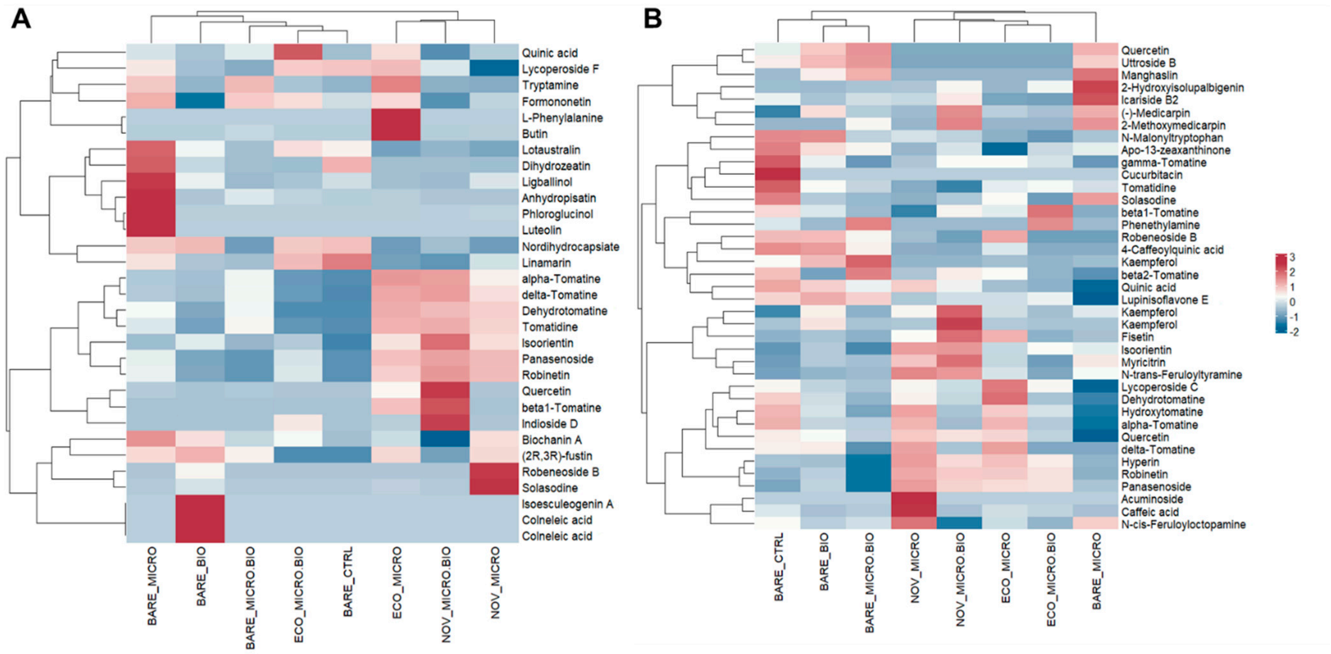

Figure S5. Heat map and dendrograms obtained by comparing the abundance of putatively identified metabolites that were statistically different in each treatment. The range of colors from blue to red shows how the intensities of the different compounds vary from the least abundant to the most abundant respectively. **A**: Abundance of significant identified metabolites for each treatment in berries samples. **B**: Abundance of significant identified metabolites for each treatment in berries samples. Each raw represents a metabolite, while data for each treatment is presented in columns. The heat map was developed using pheatmap package in R.
